# A Multidimensional Approach to Cereal Caryopsis Development: Insights into Adlay (*Coix lacryma-jobi* L.) and Emerging Applications

**DOI:** 10.3390/plants15020320

**Published:** 2026-01-21

**Authors:** Xiaoyu Yang, Jian Zhang, Maohong Ao, Jing Lei, Chenglong Yang

**Affiliations:** 1 Institute of Crop Science, Chinese Academy of Agricultural Science, Beijing 100081, China; 2 Guizhou Institute of Subtropical Crop, Guiyang 550006, China; nktzhangjian@163.com (J.Z.); aomaohong@126.com (M.A.); leijing16@sina.cn (J.L.); yangchenglong208@163.com (C.Y.); 3 Ministry of Agriculture and Rural Affairs Key Laboratory of Crop Genetic Resources and Germplasm Innovation in Karst Region, Guiyang 550006, China; 4 Guizhou Key Laboratory of Biotechnology Breeding for Special Minor Cereals, Guiyang 550006, China

**Keywords:** adlay, micro-CT, AI-assisted imaging analysis, multi-omics

## Abstract

Adlay (*Coix lacryma-jobi* L.) stands out as a vital health-promoting cereal due to its dual nutritional and medicinal properties; however, it remains significantly underdeveloped compared to major crops. The lack of mechanistic understanding of its caryopsis development and trait formation severely constrains targeted genetic improvement. While transformative technologies, specifically micro-computed tomography (micro-CT) imaging combined with AI-assisted analysis (e.g., Segment Anything Model (SAM)) and multi-omics approaches, have been successfully applied to unravel the structural and physiological complexities of model cereals, their systematic adoption in adlay research remains fragmented. Going beyond a traditional synthesis of these methodologies, this article proposes a novel, multidimensional framework specifically designed for adlay. This forward-looking strategy integrates high-resolution 3D phenotyping with spatial multi-omics data to bridge the gap between macroscopic caryopsis architecture and microscopic metabolic accumulation. By offering a precise digital solution to elucidate adlay’s unique developmental mechanisms, the proposed framework aims to accelerate precision breeding and advance the scientific modernization of this promising underutilized crop.

## 1. Introduction

Over recent decades, crop science worldwide has shifted from a narrow focus on caloric sufficiency to a more comprehensive paradigm that integrates precision nutrition, human health, and functional food systems [1]. In this new paradigm, cereals are increasingly assessed not only by their yield potential but also by their nutritional profiles and health-promoting properties [2]. Within this context, long-neglected minor cereal crops with medicinal–edible attributes are regaining attention, as they offer unique opportunities to bridge staple food production with bioactive nutrition relevant to chronic disease prevention [3].

**Figure 1:**
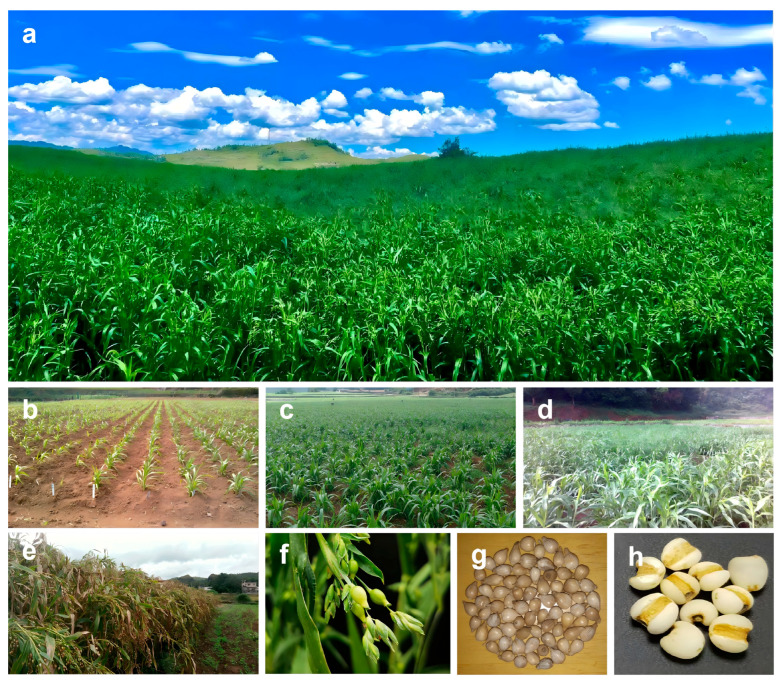
Developmental stages and morphology of adlay (*Coix lacryma-jobi* L.). (**a**) Field view at an adlay planting base in Xingyi, Guizhou Province, China; (**b**) Seedling stage; (**c**) Jointing stage; (**d**) Booting stage; (**e**) Maturity; (**f**) Close-up of spikelets; (**g**) Hulled coix seeds; (**h**) Polished kernels. Scale bars are not applicable as images are for illustrative purposes.

Adlay (*Coix lacryma-jobi* L.), a distinctive medicinal food crop within the Poaceae family, is distinguished among minor cereals by its remarkable nutritional and functional values [4]. Phylogenetic analysis confirms that adlay, maize (*Zea mays*), and sorghum (*Sorghum bicolor*) are closely related C4 crops within the tribe Andropogoneae [5]. As one of the primary centers of domestication, China has cultivated this crop for thousands of years [5,6]. China, particularly Guizhou Province, is currently the world’s leading producer, with an annual output exceeding 280,000 tons [7]. Figure 1 presents an overview of adlay, illustrating representative field stands, the major developmental stages, and the morphology of spikelets together with dehulled and polished kernels. Unlike major cereals primarily optimized for starch accumulation, adlay grains are rich in lipids, high-quality proteins, polysaccharides, and diverse bioactive metabolites with immunomodulatory and antitumor activities, highlighting adlay as a high-value functional crop for the health and wellness industry [4,8]. However, despite its long-recognized nutritional and medicinal value, the biological processes underlying adlay grain quality remain poorly understood compared to the extensive knowledge available for main food crops, such as rice, maize and wheat [9,10,11]. In particular, adlay utilization and productivity are constrained by unfavorable traits, including excessive kernel hardness, prolonged cooking time, inferior palatability, and inefficient grain filling leading to yield gaps [12,13]. These limitations likely stem from uncoordinated structural morphogenesis and metabolic deposition during caryopsis development [14,15,16,17], the regulatory networks of which have yet to be comprehensively elucidated in adlay.

This contrast highlights a major obstacle facing many underutilized crops. Sustained research investment in major cereals such as maize, rice, and wheat has yielded high-quality reference genomes, comprehensive mutant collections, and advanced phenotyping platforms, enabling systematic trait improvement through well-established genetic and breeding pipelines [18]. In contrast, adlay is a high-value but underutilized crop that lacks comparable genomic and genetic resources [5]. As a result, advances in adlay improvement have been constrained more by historical underinvestment in foundational research than by inherent agronomic limitations. Evidence from model cereals demonstrates that grain quality traits arise from the coordinated interaction between tissue architecture and metabolic processes, rather than from the action of individual genes in isolation [17]. Comparable interactions between structural and metabolic factors are likely to regulate adlay caryopsis development; however, due to the lack of detailed characterization of its tissue organization and spatially resolved metabolic partitioning, these mechanisms remain speculative, thereby impeding targeted strategies for improving grain quality.

Recent technological advances enable high-resolution examination of the relationships between internal plant structure and metabolic function without requiring extensive prior genomic resources [19]. Key among these are three-dimensional non-destructive phenotyping, spatially resolved omics, and artificial intelligence-assisted analysis. Micro-computed tomography (micro-CT), for example, supports three-dimensional in silico reconstruction of the cereal caryopsis and quantitative evaluation of vascular architecture, tissue organization, and spatial variation in grain filling [14,15]. Spatial transcriptomics and metabolomics, in turn, map gene expression and metabolite distributions to specific tissue locations, avoiding the averaging effects of bulk analyses that mask cellular- and tissue-level differences [9,10].

These technologies enable accelerated improvement of underutilized crops such as adlay through direct integration of high-dimensional phenotypic and chemotypic data. Here, we propose a closed-loop multidimensional framework that combines three-dimensional non-destructive phenotyping, spatial omics, and AI-driven analysis in an iterative cycle. Its novelty stems from the coordinated application of these tools to repeatedly connect structure, metabolism, and agronomic traits [20]. By addressing the central challenge of adlay improvement—coordinating internal structural development with the accumulation of multiple bioactive compounds—the framework seeks to shift grain enhancement from empirical selection to precision design and provide a general model for other medicinal-edible minor cereals.

## 2. Three-Dimensional Imaging Technologies for Structural Analysis of Cereal Caryopses

Cereals are staple foods worldwide, and their grain quality, processing suitability, germination potential, and stress tolerance depend heavily on the internal structure, spatial arrangement, and physical properties of the caryopsis [21]. Efficient and accurate analysis of these structures is, therefore, crucial for genetic studies and crop breeding. Recent progress in phenomics has shifted the focus from traditional two-dimensional qualitative assessments to quantitative three-dimensional characterization of plant organs. In this regard, non-destructive 3D imaging techniques, especially micro-computed tomography (micro-CT), stand out by overcoming the limitations of conventional methods while maintaining tissue integrity [22]. When paired with AI-driven image analysis tools—such as segmentation, registration, and morphometric measurements—these approaches enable high-throughput and precise 3D phenotyping, laying a solid foundation for modern cereal structural research [23].

### 2.1. Micro-CT-Based Non-Destructive 3D Imaging

Conventional histological techniques, such as paraffin embedding and semi-thin sectioning, have historically underpinned caryopsis analysis. However, these methods are characterized by inherent limitations: they are labor-intensive, susceptible to fixation-induced artifacts, and restricted to two-dimensional (2D) planes that preclude accurate three-dimensional (3D) spatial reconstruction [21,24]. While electron (SEM/TEM) and confocal microscopy offer superior resolution, their utility in large-scale crop phenotyping is constrained by destructive sample preparation, shallow penetration depths (typically <50 μm), and the frequent requisite for fluorescent labeling [25,26]. In contrast, micro-CT facilitates non-destructive volumetric reconstruction by mapping differential X-ray attenuation coefficients based on tissue density [22]. This capability allows for the precise segmentation of internal compartments and the preservation of specimen integrity for subsequent biochemical assays [21]. Furthermore, protocol optimizations, such as phosphotungstic acid contrast enhancement, have significantly improved the resolution of hydrated soft tissues in cereal grains [27].

However, micro-CT applications involve a crucial compromise: higher spatial resolution (smaller voxel size) provides finer detail but reduces field of view and throughput, while lower resolutions enable faster, batch scanning of whole grains [22,28]. As shown in Table 1, imaging protocols generally segregate into three resolution levels, each corresponding to distinct biological objectives. Current research in major cereals predominantly utilizes low- to medium-resolution scanning to assess yield-related morphometry or macroscopic quality defects [21,24,25,28]. Yet these resolutions often fall short for developmental questions in understudied crops like adlay.

A critical bottleneck in adlay improvement lies in elucidating the mechanism of early endosperm cellularization, a stage where nascent cell walls are typically thinner than 1 µm [26]. Standard absorption-based micro-CT at medium resolutions is insufficient for capturing such ultrastructural features [22]. Consequently, advanced imaging modalities, particularly phase-contrast tomography, are requisite to resolve these fine boundaries [26]. We therefore propose a “Leapfrog Strategy” for adlay research: rather than reiterating the exploratory low-resolution studies common in major cereals, researchers should directly adopt sub-micron and phase-contrast protocols. This approach will accelerate the quantification of micro-traits—such as endosperm porosity and embryo-to-endosperm volumetric ratios—thereby uncovering the structural basis of adlay’s superior nutritional profile and seed vigor.

### 2.2. Deep Learning-Based Analysis and Phenotypic Trait Extraction

Micro-CT generates large three-dimensional datasets that present considerable challenges for efficient and accurate segmentation and quantification of tissues and organs [30]. Traditional methods, such as thresholding, region growing, and watershed algorithms, rely on manually adjusted parameters and exhibit poor performance on images with complex morphologies or low contrast; moreover, they are time-consuming and labor-intensive [31]. Advances in deep learning, particularly convolutional neural networks, have revolutionized image segmentation. Architectures including Fully Convolutional Networks, U-Net, and DeepLab enable the learning of hierarchical features and end-to-end pixel-level prediction, substantially enhancing accuracy and throughput [29]. For instance, application of DeepLabV3+ with an Xception backbone to micro-CT images of coconut fruits and seeds has facilitated automated segmentation and non-destructive extraction of multiple agronomic and digital traits [32]. Historically, studies of cereal caryopsis structure have evolved through three phases: (i) initial dependence on destructive histological techniques, such as paraffin sectioning, which offered limited throughput and precision; (ii) the introduction of micro-CT for non-destructive three-dimensional imaging and accurate internal quantification; and (iii) the ongoing integration of micro-CT with deep learning-based segmentation, progressing towards scalable, data-driven structural phenotyping.

**Figure 2:**
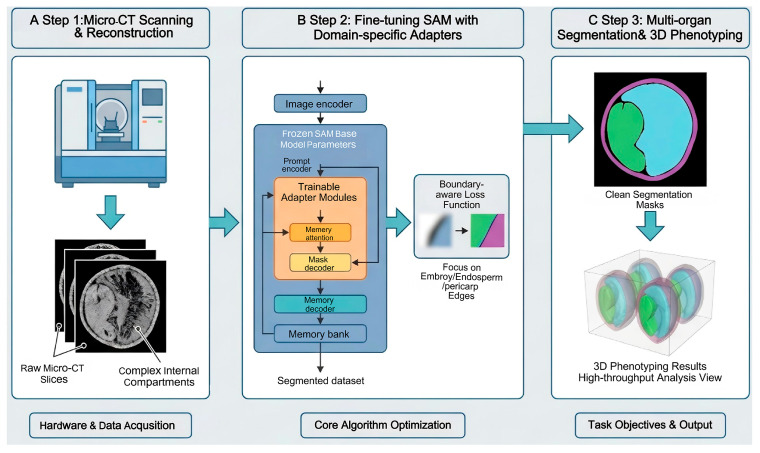
Integrated workflow of micro-CT scanning and fine-tuned segment anything model (SAM) architecture for precise cereal grain structural analysis.

While deep learning frameworks have advanced micro-CT image analysis, their implementation typically requires substantial computational expertise, posing a barrier for plant scientists. The Segment Anything Model (SAM), pre-trained on the SA-1B dataset, has demonstrated remarkable zero-shot generalization, allowing researchers to segment objects via simple prompts [33]. For crop phenotyping, SAM significantly lowers the technical threshold; users can achieve precise segmentation of diverse tissues in micro-CT images using simple point or box prompts, often without the need for additional model training [34,35]. This robustness to unseen plant structures facilitates the rapid processing of large CT datasets and supports high-throughput workflows. However, SAM is not a panacea for micro-scale caryopsis phenotyping. When applied to the complex, intricate internal compartments of adlay seeds, such as the irregular boundaries between the starchy endosperm and the scutellum, the standard “off-the-shelf” SAM often exhibits over-segmentation or fails to capture fine-grained boundaries due to the lack of domain-specific semantic knowledge [36,37]. Crucially, for the establishment of a robust, high-throughput analysis platform tailored to specific developmental stages of adlay, task-specific fine-tuning of SAM is a prerequisite. Recent studies indicate that incorporating domain-specific adapters or employing conditional tuning strategies can significantly enhance SAM’s performance on agricultural imagery [35,38,39]. Therefore, we propose a tailored workflow that integrates micro-CT scanning with a fine-tuned SAM architecture (Figure 2). This fine-tuning process bridges the gap between general-purpose vision models and the precise demands of plant structural biology.

In adlay research, a synergistic approach that leverages SAM for rapid initial segmentation followed by a fine-tuned, task-specific model for refinement holds great potential. Such a pipeline can enable the efficient extraction of multidimensional phenotypic traits, including 3D endosperm spatial distribution patterns, tissue volume ratios, and inferred hardness metrics. This methodology not only promises novel insights into the structure-function relationships governing caryopsis development and quality but also provides a refined toolkit for molecular breeding. Ultimately, it paves the way for a leapfrog strategy aimed at the synergistic improvement of both yield and nutritional quality in adlay.

## 3. Genetic Regulation and Multi-Omics Analysis of Cereal Caryopsis Development

The development of the caryopsis is the physiological cornerstone of grain yield and quality in cereals, directly impacting global food security. Recent advances in multi-omics have revolutionized our understanding, shifting the research paradigm from single gene functions to the holistic reconstruction of integrated regulatory networks. This section systematically reviews the recent progress in major cereals, tracing the logic from macro-phenotypic manifestations to micro-regulatory mechanisms, with a specialized focus on elucidating the spatiotemporal complexity of diverse grain components. Finally, we discuss how these high-dimensional insights can be leveraged to facilitate the genetic improvement and trait innovation of adlay.

### 3.1. Genetic Basis of Cereal Caryopsis Development

#### 3.1.1. Transcriptional Regulation of Storage Reserves

Caryopsis development is governed by a hierarchical genetic network wherein transcription factors (TFs) act as central hubs coordinating the spatio-temporal accumulation of storage reserves [17]. Recent advances in transcriptomics have elucidated the regulatory modules controlling the synthesis of starch and storage proteins. For instance, in maize (*Zea mays*), the bZIP transcription factor *ZmbZIP22* fine-tunes the amylose-to-amylopectin ratio by differentially regulating *GBSSIa* and other starch-synthesis genes [40]. More recently, *Opaque11* was identified as a key regulator that directly activates *ZmSSRP1* to promote starch filling, thereby determining final kernel weight [41]. Similarly, the plastidial protein OsRESR1 in rice (*Oryza sativa*) interacts with Pho1 to modulate starch granule initiation; its deficiency leads to a floury endosperm phenotype [42]. Beyond carbohydrate metabolism, proteostasis within the endoplasmic reticulum is critical for storage protein accumulation, relying on chaperones such as PDIL1-1 and BiP to prevent the formation of malformed protein bodies [43,44]. Notably, these accumulation processes are subject to evolutionary trade-offs. The broad expression of *ZmICE1a* in maize endosperm, for example, integrates storage synthesis with stress responses; its loss compromises starch content while upregulating jasmonate- and auxin-mediated defense networks [45].

#### 3.1.2. Phytohormones and Source-Sink Coordination

Phytohormones function as systemic mobile signals that coordinate the metabolic flux between source organs (leaves) and sink tissues (caryopses). Endogenous hormones, particularly abscisic acid (ABA) and auxins (IAA), orchestrate cell cycle progression and the rate of grain filling [46,47]. Crucially, signaling crosstalk between maternal tissues (pericarp/seed coat) and the filial endosperm drives caryopsis expansion. A breakthrough study by Lima et al. (2024) demonstrated that brassinosteroid (BR) signaling originating from the seed coat modulates the mechanical properties of the cell wall, thereby regulating endosperm proliferation independent of cellularization [48]. Such programmed cell death (PCD) and signaling events in maternal tissues are prerequisites for efficient nutrient unloading [49,50]. However, despite the identification of these key signaling molecules, current analytical approaches often rely on homogenates of whole grains or specific tissues. This coarse resolution fails to capture the fine-scale concentration gradients and distinct hormonal micro-environments within the developing caryopsis, which are likely responsible for the precise spatial patterning of development.

#### 3.1.3. Limitations of Current Genomic Approaches: The “Position Effect” Blind Spot

Population genomics and genome-wide association studies (GWAS) have successfully cloned major quantitative trait loci (QTLs) controlling grain size and quality. Examples include the GS2/OsGRF4 module in rice [51], the *OsCDKF3*-mediated phosphorylation pathway [52], and the *GW2* locus associated with improved nutritional quality of rice [53], and the *Wx* gene deletion determining the glutinous phenotype in adlay (*Coix lacryma-jobi* L.) [54]. However, these approaches predominantly focus on global gene expression levels. Transcriptome-based co-expression analyses, such as WGCNA in barley, have further linked hub genes like *HvENO1* to starch metabolism [55]. Nevertheless, gene mining alone offers limited insight into the “position effect”—the phenomenon where specific storage components accumulate preferentially in distinct spatial layers (e.g., aleurone vs. starchy endosperm). The current understanding of how genetic networks interpret positional cues to establish this chemical heterogeneity remains fragmentary. Consequently, a shift from purely genetic characterization to a multidimensional spatial framework is essential to decipher the complex architecture of caryopsis development and to enable a leapfrog strategy for orphan crops like adlay.

### 3.2. Advances in Multi-Omics Studies of Caryopsis Development

The advent of high-throughput omics technologies has revolutionized the study of cereal grain development by enabling a paradigm shift from single-layer observations to multidimensional network reconstruction. By integrating transcriptomic and metabolomic datasets, researchers can now elucidate the regulatory architectures governing nutrient accumulation. Furthermore, the emergence of spatial omics provides a novel avenue to bridge the gap between genotype and phenotype by preserving spatial context. This section outlines these cutting-edge methodologies and proposes a strategic framework for their application in adlay (*Coix lacryma-jobi* L.).

#### 3.2.1. Transcriptome-Metabolome Association Analysis

Integrated transcriptomic and metabolomic analysis serves as a powerful tool for decoding complex agronomic traits by linking dynamic gene expression profiles with metabolite flux patterns [56]. Methodologically, weighted gene co-expression network analysis (WGCNA) facilitates the identification of hub regulators within large-scale datasets. For instance, Zhang et al. (2016) employed WGCNA to reconstruct the lignan biosynthesis network in Isatis indigotica, identifying *4CL3* as a critical regulatory node [57]. Similarly, in cereal quality research, Jiang et al. (2024) integrated time-series transcriptomics with wide-target metabolomics to reveal that the transcription factor *OsbZIP10* regulates flavonoid pathways in rice, thereby influencing nutritional quality [58].

A holistic understanding of endosperm development requires systematic analysis of high-resolution transcriptomic and metabolic dynamics. In maize, research has elucidated the coordinated regulation of sucrose transport, unloading, and starch synthesis, highlighting the central role of sugar signaling in grain formation [59]. Similarly, a multi-omics study on a barley tocotrienol-deficient mutant revealed that the loss of this metabolite disrupts redox homeostasis in the endosperm, reprograms sugar metabolism, and ultimately compromises starch accumulation [60]. These findings illustrate that phenotype formation is driven by synchronized gene-metabolite networks, a concept highly relevant to understanding the biosynthesis of functional components in adlay.

#### 3.2.2. Spatial Omics: Bridging the Genotype-Phenotype Gap

While bulk omics offer a comprehensive overview of cellular activities, they inherently obscure the spatial heterogeneity present in complex tissues. Spatial omics technologies have emerged as a transformative solution, enabling the in situ profiling of molecular signatures while preserving histological integrity [61,62]. In cereal grains, these techniques are increasingly being applied to elucidate the compartmentalization of nutritionally significant compounds. For example, combined X-ray microscopy and Raman spectroscopy in wheat revealed significantly higher concentrations of mineral elements such as phosphorus, potassium, and magnesium in the aleurone layer compared to the starchy endosperm [63]. In rice, spatial analyses have confirmed the aleurone layer as a primary site for lipid and phenolic compound accumulation, contrasting with the starch-dominated endosperm [64]. This spatially defined metabolic specialization underpins critical aspects of grain nutritional quality and processing performance.

Recent advances have pushed spatial resolution to cellular and subcellular levels, enabling the dissection of region-specific gene regulatory networks during development [62,65]. In maize endosperm, an integration of spatially resolved gene expression and chromatin accessibility data decoded the differentiation network, revealing distinct regulatory mechanisms between zein and starch biosynthesis pathways [66]. Subsequently, an integrated ATAC-seq and RNA-seq analysis of early kernels mapped dynamic chromatin accessibility landscapes, identifying stage-specific distal accessible chromatin regions (dACRs) and revealing potential co-regulatory modules shared between the maternal chalaza fusion zone and the filial basal endosperm transfer layer (BETL) [67]. Expanding this functional dimension, spatial transcriptomics was utilized to uncover the sucrose post-phloem transport pathway, identifying specific *SWEET* transporter expression in the placento–chalazal region that governs sink strength [68]. Beyond nutrient partitioning, high-resolution analysis has been extended to the maize embryo, resolving the transcriptional heterogeneity between the scutellum and embryonic axis, which offers a blueprint for dissecting lipid storage compartments in high-oil cereals [69].

For adlay (*Coix lacryma-jobi* L.), spatial omics technologies provide an unprecedented opportunity to unravel the coordinated biosynthesis of its diverse bioactive compounds [70,71]. Future studies could integrate these approaches to simultaneously pinpoint the sites of synthesis and accumulation of key components, including lipids (potentially enriched around the embryo) [72], polysaccharides (predominantly accumulating in the endosperm), and phenolic compounds (mainly synthesized near the seed coat) [73]. Deciphering this spatially resolved “metabolic map” will be essential for informing targeted, step-change breeding strategies aimed at enhancing the nutritional and medicinal value of adlay caryopses.

### 3.3. Core Technical Challenges in Cereal Developmental Studies

Unraveling the complex genetic regulatory networks (GRNs) that govern cereal caryopsis development requires the tight integration of high-dimensional multi-omics data with precisely resolved phenotypic metrics. However, current research is hindered by a fundamental scale disparity between the single-cell resolution of modern genomics and the coarse, macroscopic nature of traditional phenotyping approaches. While genomic data now achieve nucleotide-level precision, phenotypic datasets remain plagued by heterogeneity, inconsistent annotation standards, and low dimensionality. In genetic mapping studies such as genome-wide association studies (GWAS), this data asymmetry severely limits the statistical power needed to identify quantitative trait loci (QTLs) underlying subtle internal structures [74,75]. Consequently, the absence of high-throughput, structurally resolved phenotypic data has emerged as the primary bottleneck in bridging the genotype-to-phenotype gap.

Compounding this data heterogeneity is the technical challenge of acquiring high-fidelity structural information, which is particularly pronounced during the early stages of caryopsis development. In cereals such as adlay (*Coix lacryma-jobi* L.), early-stage grains exhibit high water content and low density contrast, posing substantial challenges for non-destructive imaging. Conventional X-ray micro-computed tomography (micro-CT) often fails to differentiate the developing embryo from the fluid-filled endosperm in fresh, hydrated tissues. To resolve these internal compartments, researchers must rely on contrast-enhancing agents and dehydration protocols [27]. Although these approaches enable structural visualization, they are invasive and labor-intensive, thereby precluding high-throughput phenotyping of large breeding populations. This throughput limitation thus impedes the large-scale identification of genes governing critical early developmental processes, including cell differentiation and storage product accumulation [76].

Moreover, even when high-resolution 3D images can be obtained, converting them into biologically meaningful measurements for genetic analysis remains a major computational hurdle. Gene regulation is highly tissue-specific: the genes that control the aleurone layer are very different from those that regulate the starchy endosperm. However, extracting these tissue-level phenotypes is still limited by the performance of current segmentation methods. Foundation models such as the Segment Anything Model (SAM), although effective for general object detection, often struggle to delineate the unclear and continuous boundaries typical of plant microstructures, including the testa and pericarp [35]. Without accurate semantic segmentation, it is difficult to quantify tissue-specific traits and reliably align them with spatial transcriptomic profiles [20,62]. Therefore, addressing these challenges, from imaging constraints to segmentation limitations, is crucial for building accurate multi-dimensional models that can support “leapfrog” design breeding in adlay.

## 4. Application Prospects of Multi-Dimensional Analytical Technologies in Adlay Research

### 4.1. Current Research Status and Critical Bottlenecks

Research on adlay (*Coix lacryma-jobi* L.) has achieved significant milestones, establishing a solid foundation in genomics, cultivation physiology, and phytochemistry. The release of the reference genome [5] and the characterization of key traits, such as floral sterility [77] and inflorescence architecture [78], have marked a transition toward functional genomics. Concurrently, agronomic protocols optimizing nitrogen management [79], sowing dates [80], and altitudinal adaptation [81] have been well-defined. In phytochemistry, efficient extraction and pharmacological validation of bioactive constituents, including lipids [72], polysaccharides [82], coixol [83], and phenolics [8,84], have reached a mature stage.

However, a fundamental disparity remains: while the chemical composition of adlay is well characterized, the spatiotemporal mechanism of caryopsis development remains a blackbox. Current understanding largely relies on seminal but traditional histological studies [85], which, due to their destructive nature, capture only static snapshots and fail to reconstruct continuous developmental trajectories. Unlike major cereals where 4D micro-structural dynamics and spatial metabolic gradients are increasingly resolved [14,68], adlay research still lacks high-resolution in situ data. The inability to non-destructively visualize endosperm filling dynamics or precisely map the spatial co-localization of genes and metabolites restricts the transition from experience-based to precision design breeding. Bridging this gap requires an urgent paradigm shift toward multi-dimensional analytics.

### 4.2. Future Directions: A Leapfrog Strategy via Multi-Dimensional Integration

**Figure 3:**
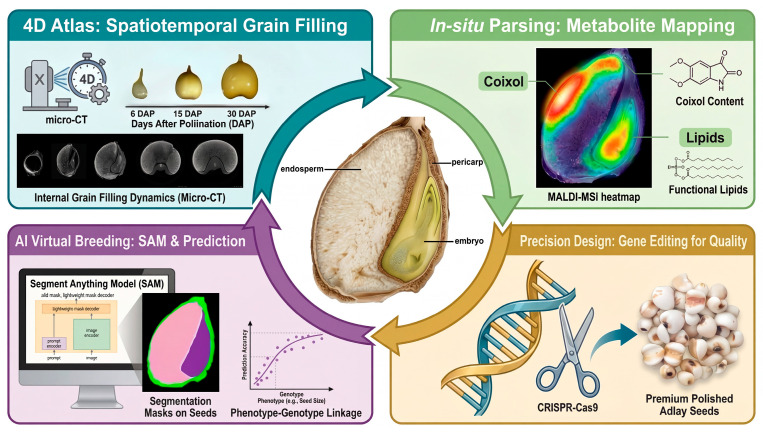
A Leapfrog Strategy for adlay precision design through multi-dimensional integration of phenomics, spatial omics, and AI.

To overcome existing bottlenecks, future research must adopt a “Leapfrog Strategy” that integrates high-throughput phenomics, spatial multi-omics, and artificial intelligence, as illustrated in our proposed framework (Figure 3). The roadmap envisions a shift from analyzing homogenized tissue samples to resolving cellular heterogeneity within the caryopsis. By coupling structural phenotyping with molecular profiling, researchers can construct a holistic “Structure–Metabolism–Gene” regulatory network. This multi-dimensional framework will support three priority initiatives aimed at revolutionizing adlay improvement:

Project 1: The 4D Adlay Caryopsis Atlas (Spatiotemporal Morphogenesis)

The first priority is to establish a four-dimensional (3D space + time) developmental atlas. Utilizing non-destructive technologies such as Synchrotron Radiation X-ray Micro-computed Tomography (SR-μCT) or high-resolution micro-CT [21,26], research should focus on the in vivo tracking of grain filling dynamics. This approach will allow for the precise quantification of endosperm cellularization, starch granule packing efficiency, and the spatial progression of programmed cell death (PCD) [16,64] without distinct seed destruction. Such an atlas will resolve long-standing ambiguities regarding the timing of aleurone layer differentiation and the vascular transport pathways limiting nutrient accumulation [50], providing morphological markers for high-yield breeding.

Project 2: In situ Pathway Parsing (Spatial Multi-Omics)

Addressing the complexity of bioactive compound accumulation, this initiative proposes the use of spatial transcriptomics and Matrix-Assisted Laser Desorption/Ionization Mass Spectrometry Imaging (MALDI-MSI) to map metabolic pathways at the tissue level [9,62]. Unlike bulk RNA-seq, these technologies can visualize the specific spatial zones responsible for the synthesis of lipids versus coixol [72,83]. By overlaying gene expression maps with metabolite distributions [68,69], researchers can decouple the synergistic regulatory networks governing quality traits, identifying precise genetic targets for metabolic engineering to enhance therapeutic value.

Project 3: AI-Assisted “Virtual Breeding” (Predictive Phenomics)

The ultimate goal is to translate micro-phenotypic data into selection efficiency. Leveraging advancing computer vision models, such as the “Segment Anything Model” (SAM) [38,40], deep learning algorithms can be trained to automatically segment and quantify internal seed features (e.g., embryo-to-endosperm ratio, porosity, and tissue density) from micro-CT images [23,29]. This will enable the development of a “Virtual Selection” platform where the chemical quality and milling yield of breeding lines can be predicted solely based on non-destructive internal morphological scans [32,75]. Such a system would dramatically accelerate the screening of germplasm resources, bypassing the labor-intensive wet-chemistry assays currently limiting breeding throughput [74].

The convergence of data from these projects will enable the construction of predictive, multiscale models of caryopsis development. Ultimately, integrating these insights with modern breeding techniques like CRISPR-Cas9 [86] and genomic selection [18] will transition adlay breeding from phenotypic selection to precision design. This integrated strategy promises synergistic gains in yield, nutritional quality, and medicinal value, establishing a new paradigm for the improvement of underutilized functional cereals.

## 5. Conclusions

In this study, we present a robust multidimensional framework that fuses micro-CT 3D imaging, spatial omics, and AI-driven analysis. By moving beyond the limitations of traditional 2D slicing, this approach allows us to visualize the “hidden” interplay between internal structural changes and medicinal compound accumulation during Coix development. We have effectively bridged the gap between macroscopic physical traits and microscopic gene expression, offering a precise digital toolkit to decode the developmental biology of this complex crop.

The implications of this strategy reach well beyond the Coix industry. While it directly addresses bottlenecks like unstable quality and breeding inefficiencies, it also serves as a replicable blueprint for modernizing the study of other underutilized Nutri-Cereals. As global agriculture shifts focus from simple volume to “precision nutrition,” unlocking the potential of these orphan crops through such advanced technologies will be crucial for diversifying our food systems and enhancing global food security.

## Figures and Tables

**Table 1:** Spatial resolution levels used in micro-CT studies of cereal grains and their relevance to adlay breeding.

Resolution Tier (Voxel Size)	Typical Applications and Traits	Example Crops and Studies	Advantages	Limitations	Relevance to Adlay (Leapfrog Strategy)
Low/Macro (~20–80 µm)	Whole-kernel morphometry: volume, density, husk/pericarp thickness, overall shape.	Maize (high-throughput single-kernel volumes/densities [28]); Sorghum grain structure [21]; Wheat grain morphology [14].	High throughput; batch scanning possible; suitable for large samples.	Cannot resolve internal tissues or fine defects.	Yield traits: Rapid, non-destructive screening of husk thickness and kernel-to-husk ratio for improved edible yield.
Medium (~5–15 µm)	Quality and internal defects: chalkiness, fissures, embryo morphology, insect damage, plumpness.	Rice chalkiness (optimal ~5 µm [25]); Maize plumpness and internal cracks [24]; Pest detection in rice [22].	Good balance of detail and speed; effective for mature grain quality assessment.	Limited for cellular-level structures or early development.	Seed quality/health: Detection of internal defects or pests without dehulling; embryo vigor screening.
High/Sub-micron (<5 µm, often phase-contrast)	Cellular microstructure: starch granule packing, aleurone layer, cell wall formation, early endosperm cellularization.	Maize developing seeds (phase-contrast [26]); Foxtail millet grain structure [29]; Wheat microstructural evolution [14].	Resolves fine cellular boundaries and developmental dynamics.	Small field of view; long scan times; high data volume.	Developmental mechanisms: Essential for visualizing early endosperm cellularization and nutrient pathways—directly adopting advanced protocols to bypass lower-resolution stages used in other cereals.

## Data Availability

The original contributions presented in this study are included in the article. Further inquiries can be directed to the corresponding author.
